# Publicação Científica na Era da Inteligência Artificial: Integridade e Responsabilidade

**DOI:** 10.36660/abc.20260338

**Published:** 2026-07-16

**Authors:** Mário Martins Oliveira, Gláucia Maria Moraes de Oliveira

**Affiliations:** 1 Hospital de Santa Marta ULS. S José Lisboa Portugal Hospital de Santa Marta; ULS. S José, Lisboa – Portugal; 2 Universidade Federal do Rio de Janeiro Rio de Janeiro RJ Brasil Universidade Federal do Rio de Janeiro, Rio de Janeiro, RJ – Brasil

**Keywords:** Inteligência Artificial, Comunicação e Divulgação Científica, Ética na Publicação Científica, Políticas Editoriais

A inteligência artificial (IA) está transformando o processo científico em todos os seus estágios, tornando-se uma ferramenta inovadora, usada em âmbito global, que influencia a geração de pesquisa, o planejamento experimental, a análise de dados, a escrita e toda a complexa dinâmica de *peer review* e divulgação de resultados.

À medida que toda a comunidade científica incorpora a IA como parte do processo de pesquisa para aprimoramento da eficiência, o desenvolvimento de regulação e algoritmos adequados para monitorar a qualidade, a veracidade e a integridade da ciência de dados torna-se um tópico crítico de discussão no que se refere ao papel fundamental da comunicação científica.

O potencial das tecnologias assistidas por IA para auxiliar pesquisadores a trabalhar com eficiência, quando usadas de maneira responsável, oferece suporte personalizado para algumas tarefas, como a organização do conteúdo de manuscritos, a análise de dados, a geração de texto, o refinamento da linguagem e a legibilidade.^1^ A IA aprimora a linguagem e a análise de dados, mas seu uso levanta questões éticas quanto à confecção do texto à medida que a submissão de manuscritos com conteúdo gerado por IA aumenta.

Ampla adoção de ferramentas de IA é esperada. Portanto, as revistas científicas atualmente fornecem recomendações de melhores práticas para a utilização de IA e aplicam ferramentas de detecção para identificar seu uso excessivo na preparação de um manuscrito.^2^

Deve-se utilizar um fluxo de trabalho estruturado e automatizado como um teste exploratório inicial para conferir a adequação da formatação, além da falta de dados ou ficheiros. O próximo passo, mesmo antes da avaliação pelo editor, é a checagem de plágio, a duplicação de submissão (não tão incomum), a manipulação de imagem, as anomalias estatísticas e a clareza de linguagem. Essa triagem com IA auxilia os editores por meio da sinalização de riscos e preocupações. Além disso, a IA pode apoiar o trabalho dos editores através da sugestão de revisores com experiência em áreas específicas ou com artigos similares publicados.

Editoras como Elsevier, Wiley ou Taylor & Francis atualmente fornecem diretrizes para o uso da IA na preparação de manuscritos, mas solicitam a divulgação do nome da tecnologia assistida por IA utilizada além da extensão de sua aplicação.^3^

Aceita-se que ferramentas assistidas por IA ajudem os pesquisadores durante o longo caminho da ciência, além de poderem atuar como uma forte contribuição para auxiliar as empresas editoras e os editores na identificação de dependência excessiva, uso não declarado, plágio ou detecção de conteúdo impreciso durante o fluxo de trabalho de *peer review*. A expansão do uso de ferramentas de IA na publicação científica é acompanhada pelos riscos de ‘autoria através de IA’, manipulação de conteúdo, surgimento de 'fábricas de artigos científicos’ e comprometimento da credibilidade quanto à solidez da comunicação.

A comunidade científica deve tomar medidas proativas para definir limites éticos, com políticas claras quanto à qualidade das fontes de dados, a autoria, a divulgação e a transparência.^4^ A utilização de algoritmos complexos de IA levanta questões quanto a vieses, transparência e responsabilidade, requerendo o desenvolvimento de novas normas éticas para proteger a integridade científica. Entretanto, o desenvolvimento e a redação de códigos de ética não conseguem acompanhar a velocidade da evolução e da implementação da tecnologia.^5^

O editorial da PNAS (*Proceedings of the National Academy of Sciences*), de autoria de um grupo interdisciplinar de especialistas, enfatiza que a IA generativa (e.g., ferramentas como o ChatGPT) deve respeitar as normas centrais da ciência. Tem por base as recomendações das *National Academies of Sciences* para a priorização da supervisão humana, instando à criação de um Conselho Estratégico para a contínua orientação quanto ao uso da IA. São cinco princípios que garantem transparência, verificação e ética no contexto da rápida integração da IA. Tais princípios devem ser aplicados em todos os estágios da pesquisa (escrita, análise de dados, revisão por pares) e estar alinhados às políticas de publicação, como as da Elsevier, que não baniu a autoria através de IA, mas requer divulgação do uso de conteúdo gerado pela ferramenta. Esses princípios contrapõem-se a riscos, como a amplificação de vieses e a erosão da confiança, com propostas de incentivo a educação e supervisão institucional.^6^

O uso apropriado da IA não deve substituir a *expertise* humana, a supervisão ética e a responsabilidade. Ao contrário, a IA deve ser usada como uma poderosa ferramenta que contribui para melhorar a qualidade da ciência de dados e, por fim, como um auxílio tecnológico inovador para manter a confiança nos periódicos, nos editores e nas instituições.^7,8^

A hora é essa e é nossa responsabilidade disseminar um forte compromisso coletivo com os padrões de integridade da publicação científica. Políticas para regulamentar o uso da IA em universidades e grupos editoriais requerem amplo debate, aplicabilidade e validação. Recomendações claras para apoiar editoras científicas, seus editores e *peer reviwers* na condução dessa desafiadora tarefa são necessárias.

**Figura 1:**
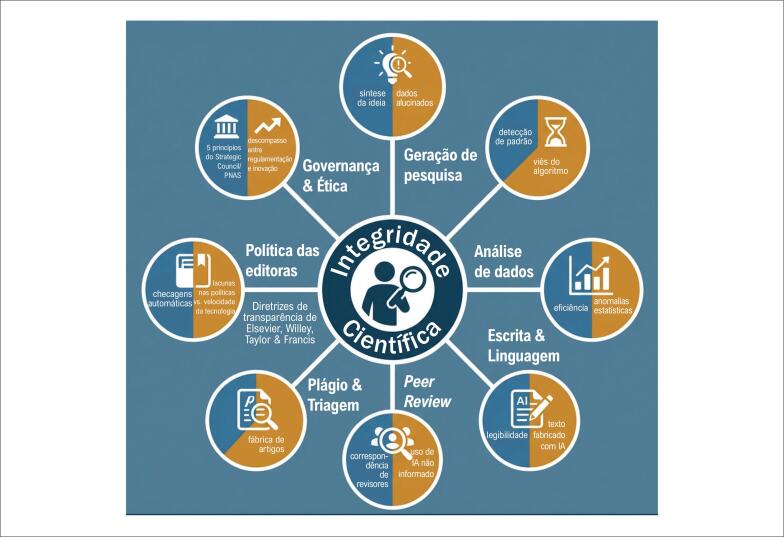
Integridade e responsabilidade na era da inteligência artificial na publicação científica. Figura criada usando a plataforma de inteligência artificial Gamma AI.

**A IA deve apoiar – e não substituir – a *expertise* humana, a supervisão ética e a responsabilidade** (Figura 1)**.**
