# Supplementary material for: Publicação Científica na Era da Inteligência Artificial: Integridade e Responsabilidade
Source: Arq Bras Cardiol. 2026 Jul 16;123(8):e20260338. [Article in Portuguese] doi: 10.36660/abc.20260338 (PMC13399034; doi:10.36660/abc.20260338)
Supplement: Translation

# Translation: Scientific Publishing in the era of Artificial Intelligence: Integrity and Responsibility

**DOI:** 10.36660/abc.20260338i

**Authors:** Mário Martins Oliveira, Gláucia Maria Moraes de Oliveira

**Affiliations:** 1 Lisboa Portugal Centro Hospitalar de Lisboa Central EPE, Lisboa – Portugal; 2 Rio de Janeiro RJ Brazil Universidade Federal do Rio de Janeiro, Rio de Janeiro, RJ – Brazil

**Keywords:** Artificial Intelligence, Scientific Communication and Diffusion, Scientific Publication Ethics, Editorial Policies

Artificial Intelligence (AI) is transforming the scientific process across all stages, making it an innovative tool, used globally, influencing research generation, experimental design, data analysis, writing, and all complex dynamics of peer review and dissemination of results.

While the entire scientific community is incorporating AI as part of the research process to enhance efficiency, the development of regulations and adequate algorithms to monitor the quality, truthfulness, and integrity of data science is becoming a critical matter of discussion, regarding the fundamental role of scientific communication.

The potential of AI-assisted technologies, when used responsibly, to help researchers work efficiently, provides tailored support for tasks, such as manuscript content organization, data analysis, text generation, language improvement, and readability.^1^ AI improves language polishing and data analysis, but it raises ethical concerns regarding text fabrication, with more submitted manuscripts showing AI-generated content.

The wide adoption of AI tools is largely expected. Therefore, scientific journals are now providing recommendations of best practices when using AI and applying detection tools to identify excessive use of AI in manuscript preparation.^2^

A structured automatic workflow must be used as an initial exploratory testing to check for adequate formatting, missing data, or files. The next step, even before the editor evaluation, is to check for plagiarism, submission duplication (not so uncommon), image manipulation, statistical anomalies, and language clarity. This AI-screening assists editors by showing flags regarding risks and concerns. In addition, AI may support editors’ work by suggesting reviewers with expertise in specific fields or with similar papers published.

Publishers like Elsevier, Wiley, or Taylor & Francis are no providing AI usage guidelines for manuscript preparation, but ask for a declaration statement, as a disclosure, regarding the name of the AI-assisted technology used and the extent of its application.^3^

It is accepted that AI-assisted tools support researchers during the long pathway of science, but can also work as a strong contributor to assist publishers and editors in identifying excessive reliance, undisclosed use, plagiarism, or inaccurate content detection during the peer review workflow. The expansion of AI tools in scientific publishing is accompanied by the risks of "AI authorship", content manipulatation, emergence of papermills, and the potential compromise of the credibility of communication.

The scientific community must take active measures to define ethical boundaries, with clear policies regarding the quality of data sources, authorship, disclosures, and transparency.^4^ The use of complex AI algorithms raises concerns about bias, transparency, and accountability, requiring the development of new ethical rules to protect scientific integrity. However, the development and writing of ethical codes cannot keep up with the pace of technology development and implementation.^5^

The PNAS (Proceedings of the National Academy of Sciences) editorial, authored by an interdisciplinary group of experts, emphasizes that generative AI (e.g., tools like ChatGPT) must not erode the core norms of science. It builds on the *National Academies of Sciences* recommendations to prioritize human oversight, urging the creation of a Strategic Council for ongoing AI guidance. The five principles ensure transparency, verification, and ethics amid AI's rapid integration and must be applied across research stages (e.g., writing, data analysis, peer review) and align with publisher's policies, such as Elsevier's, which do not ban AI authorship but require disclosure. These principles address risks, such as the amplification of biases and the erosion of trust, with proposals to promote education and institutional oversight.^6^

The appropriate use of AI should not replace human expertise, ethical oversight, and responsibility. Instead, AI should be used as a powerful tool that contributes to improving the quality of data science and, ultimately, as an innovative technological support to maintain trust in journals, editors, and institutions.^7,8^

It is our responsibility (and the time) to spread a strong collective commitment to scientific publishing integrity standards. Policies regulating the use of AI in universities and in publishing groups require extensive debate, applicability, and validation. There is a need for clear recommendations to support publishers, editors, and peer reviewers navigating this challenging scientific task.

**AI must support – not replace – human expertise, ethical oversight, and responsibility** (Figure 1).

**Figure 1:** Integrity and responsibility in the era of Artificial Intelligence in Scientific publishing. Figure created using Gamma AI artificial intelligence-powered platform.
